# Supplementary material for: GSK3α phosphorylates dynamin-2 to promote GLUT4 endocytosis in muscle cells
Source: J Cell Biol. 2022 Nov 29;222(2):e202102119. doi: 10.1083/jcb.202102119 (PMC9712776; doi:10.1083/jcb.202102119)
Supplement: Table S1 — lists plasmids used in this study. [file JCB_202102119_TableS1.docx]

**Table S1**

List of plasmids used in this study.

| Plasmid | Vector | Source |
| --- | --- | --- |
| Dyn2 | pIEx-6 | Schmid lab^#^ |
| Dyn2R465W | pIEx-6 | Chin et al., 2015 |
| Dyn2A618T | pIEx-6 | Chin et al., 2015 |
| Dyn2S619L | pIEx-6 | Chin et al., 2015 |
| Dyn2∆625 | pIEx-6 | Chin et al., 2015 |
| Dyn2S848A | pIEx-6 | In this study |
| Dyn2S848E | pIEx-6 | In this study |
| Dyn2S856A | pIEx-6 | In this study |
| Dyn2S856E | pIEx-6 | In this study |
| Bin1 | pGEX-4T-1 | In this study |
| Bin1∆SH3 | pGEX-4T-1 | In this study |
| Bin1∆PI | pGEX-4T-1 | In this study |
| N-BAR | pGEX-4T-1 | In this study |
| Endophilin | pGEX | Schmid lab^#^ |
| Bin1 | pET-30a | In this study |
| Bin1Q434X | pET-30a | In this study |
| Bin1K436X | pET-30a | In this study |
| Bin1-GFP | pEGFP-C1 | Addgene 22213 |
| Bin1Q434X-GFP | pEGFP-C1 | In this study |
| Bin1K436X-GFP | pEGFP-C1 | In this study |
| Dyn2-mCherry | pmCherry-N1 | Addgene 27689 |
| Dyn2S848A-mCherry | pmCherry-N1 | In this study |
| Dyn2S848E-mCherry | pmCherry-N1 | In this study |
| Dyn2G537C-mCherry | pmCherry-N1 | Chin et al., 2015 |
| HA-Dyn2 | pAS4.1w.Ppuro-aOn | In this study |
| HA-Dyn2S848A | pAS4.1w.Ppuro-aOn | In this study |
| HA-Dyn2S848E | pAS4.1w.Ppuro-aOn | In this study |
| HA-GLUT4-GFP | pQBI25 | McGraw lab* |
| HA-Dyn2 | pcDNA3.0-HA | In this study |
| HA-Dyn2S848A | pcDNA3.0-HA | In this study |
| GSK3A | pMT2 | Addgene 15896 |
| GSK3A-S21A | pMT2 | In this study |
| GSK3A-K148A | pMT2 | In this study |
| HA-GSK3B-S9A | pcDNA3 | Addgene 14754 |
| GSK3A-S21A-HA | pLAS2w.Ppuro | In this study |
| GSK3A-K148A-HA | pLAS2w.Ppuro | In this study |
| GSK3B-S9A-HA | pLAS2w.Ppuro | In this study |
| GSK3B-K85A-HA | pLAS2w.Ppuro | In this study |

#Dr. Sandra Schmid in the Department of Cell Biology, University of Texas Southwestern Medical Center, Dallas, TX 75390, USA

*Dr. Timothy E. McGraw in Department of Biochemistry, Weill Cornell Medical College, New York, NY 10065
