# Supplementary material for: GSK3α phosphorylates dynamin-2 to promote GLUT4 endocytosis in muscle cells
Source: J Cell Biol. 2022 Nov 29;222(2):e202102119. doi: 10.1083/jcb.202102119 (PMC9712776; doi:10.1083/jcb.202102119)
Supplement: Table S2 — lists antibodies used in this study. [file JCB_202102119_TableS2.docx]

**Table S2**

List of antibodies used in this study.

| Antigen | Host | Manufacturer and catalog number |
| --- | --- | --- |
| GLUT4 | Mouse | Cell Signaling Technology, 2213 |
| HA | Mouse | Biolegend, 901513 |
| Golgin-97 | Mouse | Molecular Probes, A-21270 |
| Na+/K+ ATPase (C464.6) | Mouse | Santa Cruz, sc-21712 |
| α-Tubulin | Mouse | Sigma-Aldrich, T6074 |
| p-Ser (16B4) | Mouse | Santa Cruz, sc-81514 |
| GSK3α | Rabbit | Cell Signaling Technology, 9338 |
| Akt | Rabbit | Cell Signaling Technology, 4691 |
| p-Akt (Ser473) | Rabbit | Cell Signaling Technology, 4060 |
| GSK3α/β | Rabbit | Cell Signaling Technology, 5676 |
| p-GSK3α/β (Ser 21/9) | Rabbit | Cell Signaling Technology, 9331 |
| p-Dyn2^S848^ (p-S848) | Rabbit | Self-generated in this study (raised against PGVP(pSer)RRPPAAPSRC epitope) |
| Bin1-PI motif | Rabbit | Self-generated in this study (raised against RKKSKLFSRLRRKKN epitope) |
| Dyn2 | Goat | Santa Cruz, sc-6400 |
