# Supplementary figures and images for: GSK3α phosphorylates dynamin-2 to promote GLUT4 endocytosis in muscle cells
Source: J Cell Biol. 2022 Nov 29;222(2):e202102119. doi: 10.1083/jcb.202102119 (PMC9712776; doi:10.1083/jcb.202102119)

Fig 1F

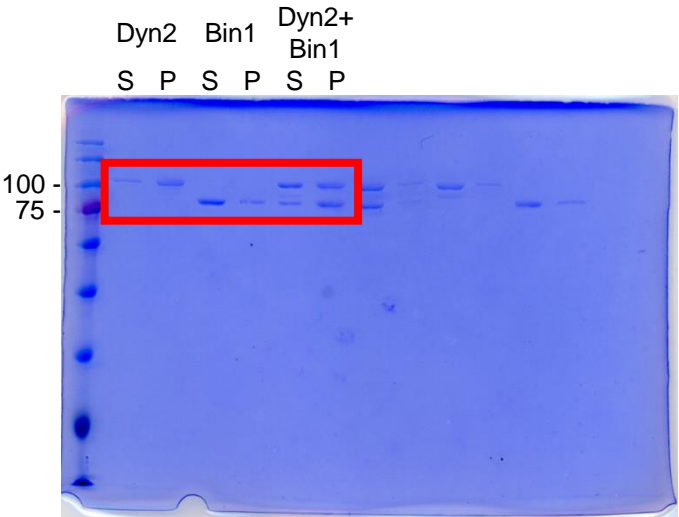

Supplement: SourceData F1 — contains original blots for Fig. 1. [file JCB_202102119_SourceDataF1.pdf]

Fig 2B

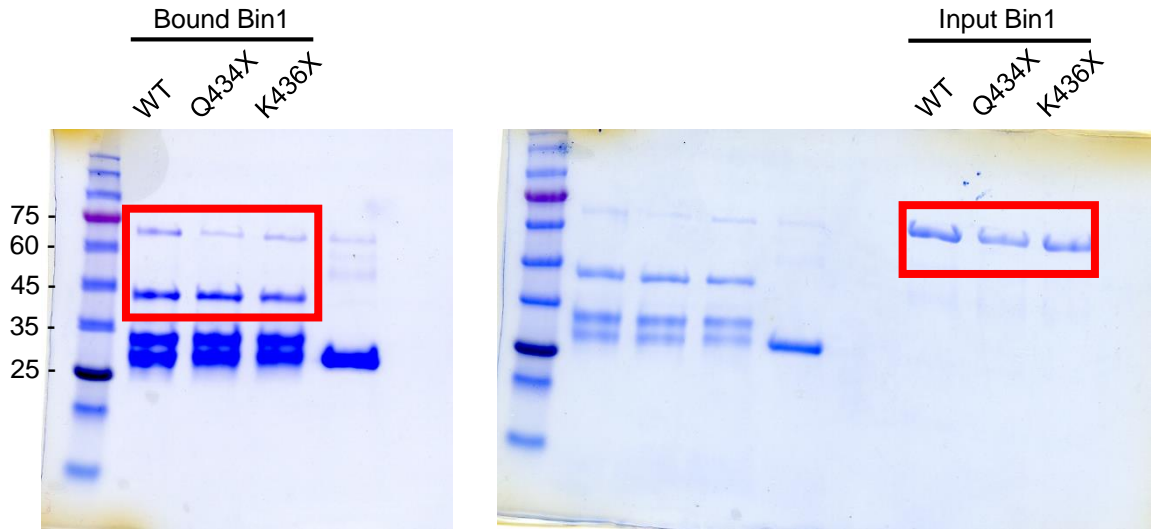

**Fig 2D**

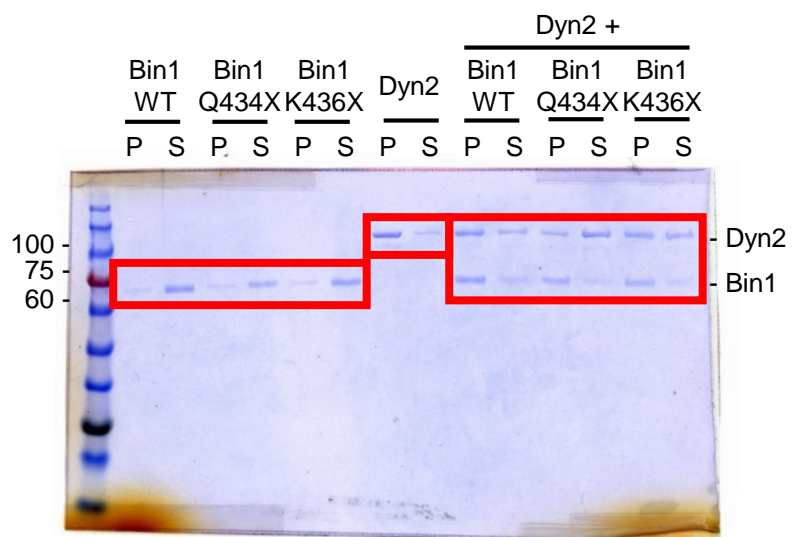

Supplement: SourceData F2 — contains original blots for Fig. 2. [file JCB_202102119_SourceDataF2.pdf]

Fig 3B

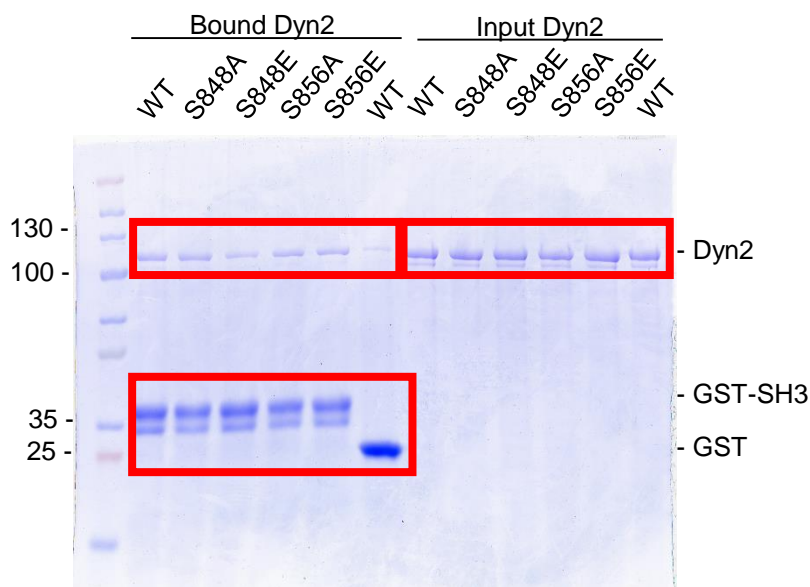

Supplement: SourceData F3 — contains original blots for Fig. 3. [file JCB_202102119_SourceDataF3.pdf]

Fig 4A

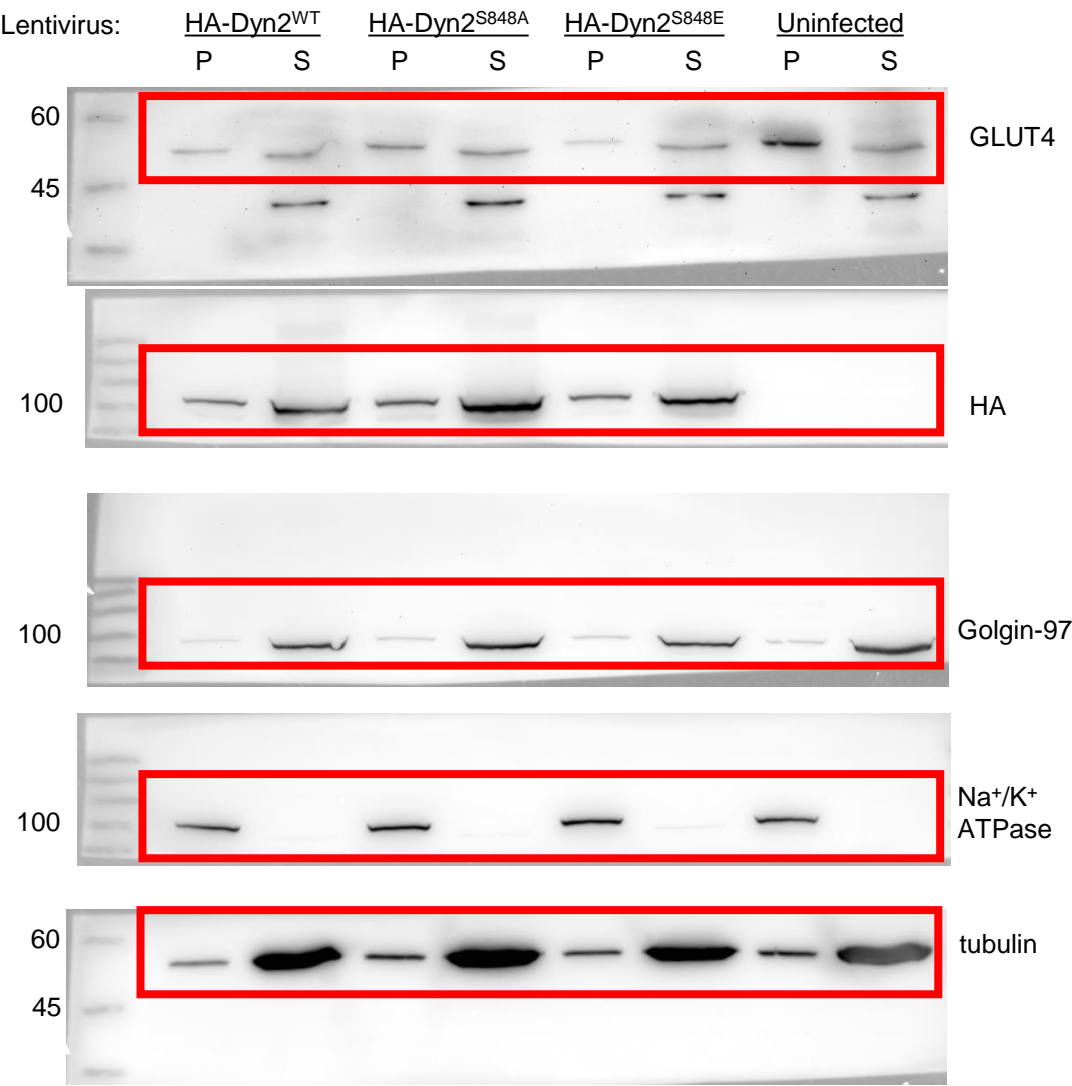

Supplement: SourceData F4 — contains original blots for Fig. 4. [file JCB_202102119_SourceDataF4.pdf]

Fig 5A

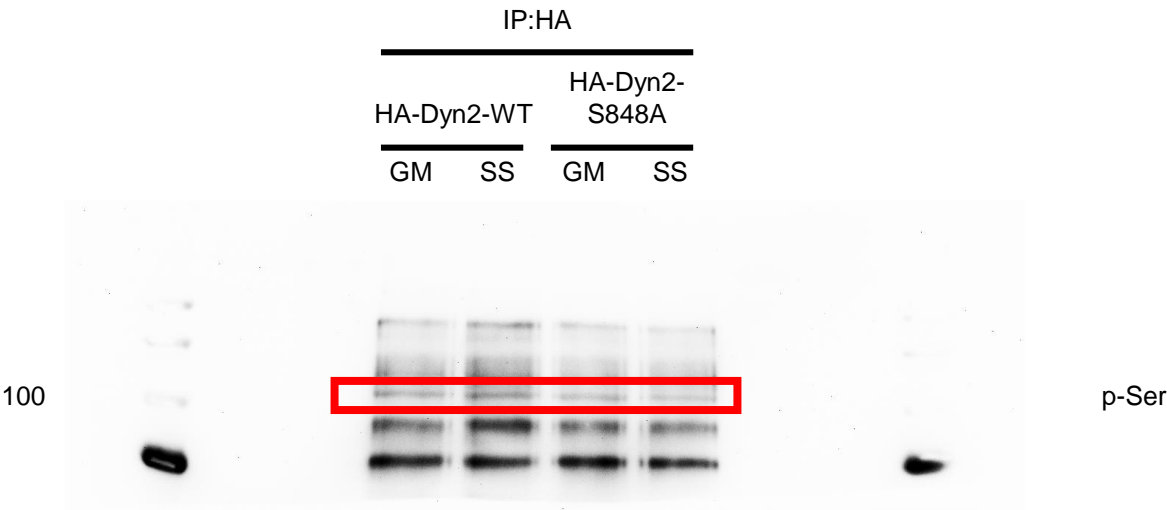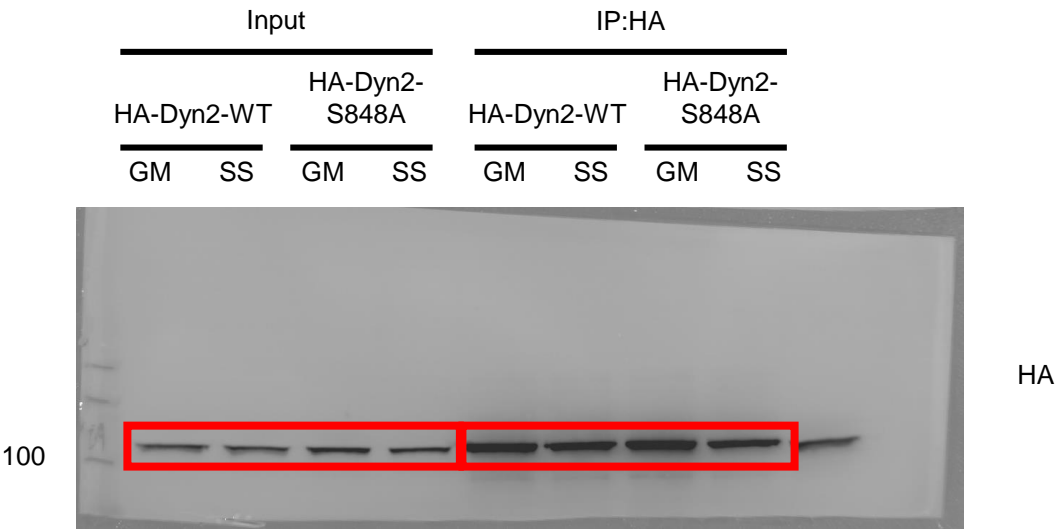

Fig 5C

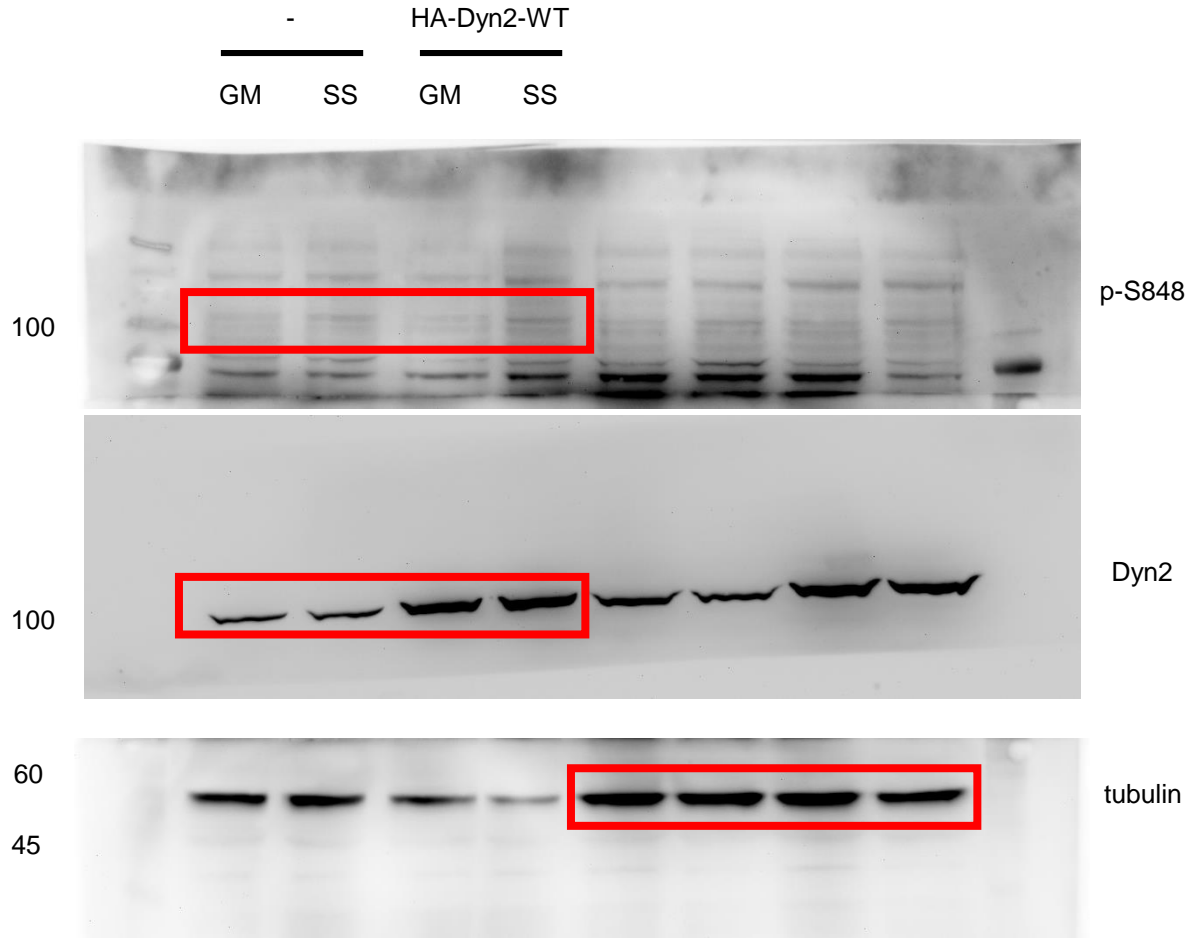

Fig 5D

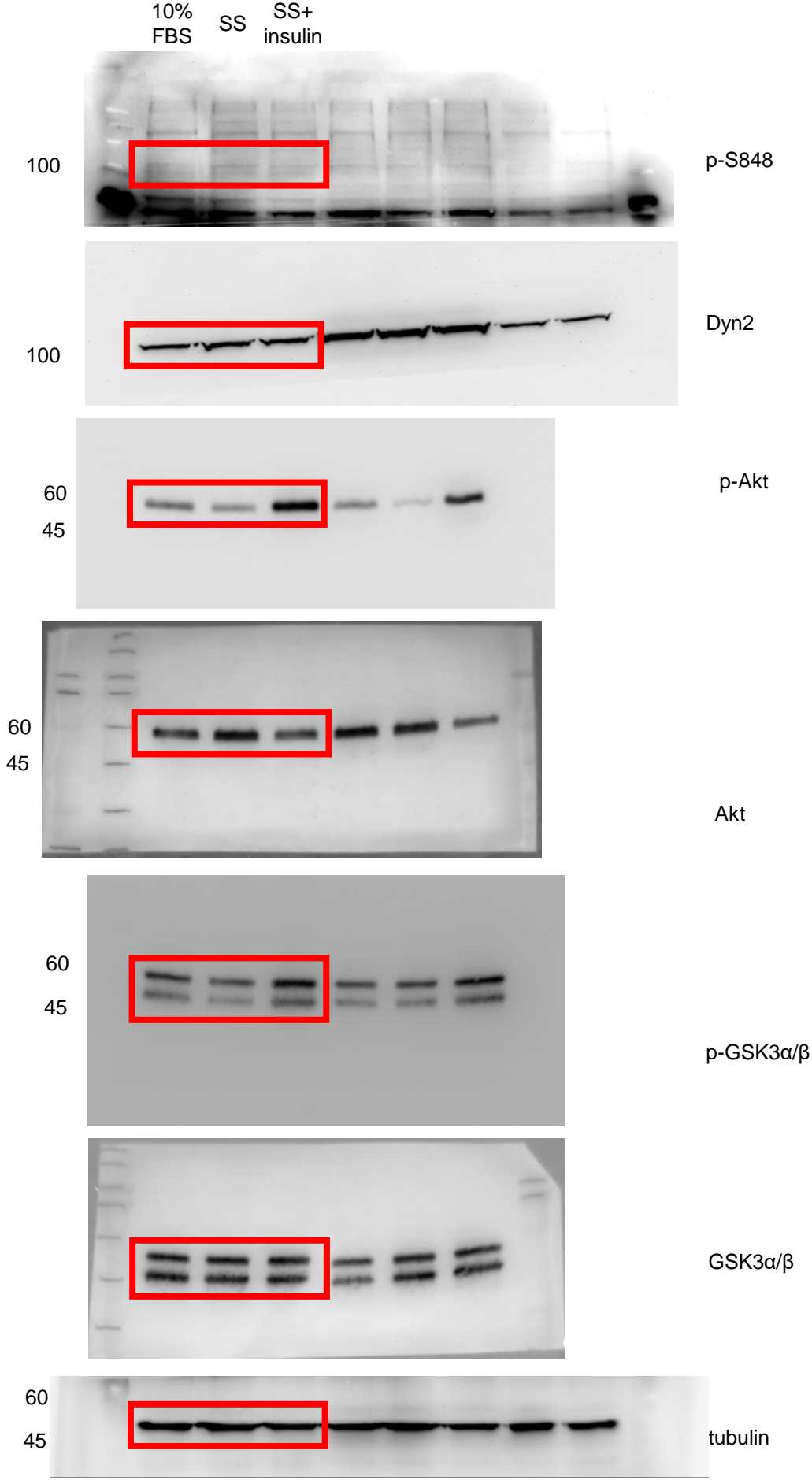

Supplement: SourceData F5 — contains original blots for Fig. 5. [file JCB_202102119_SourceDataF5.pdf]

Fig 6A

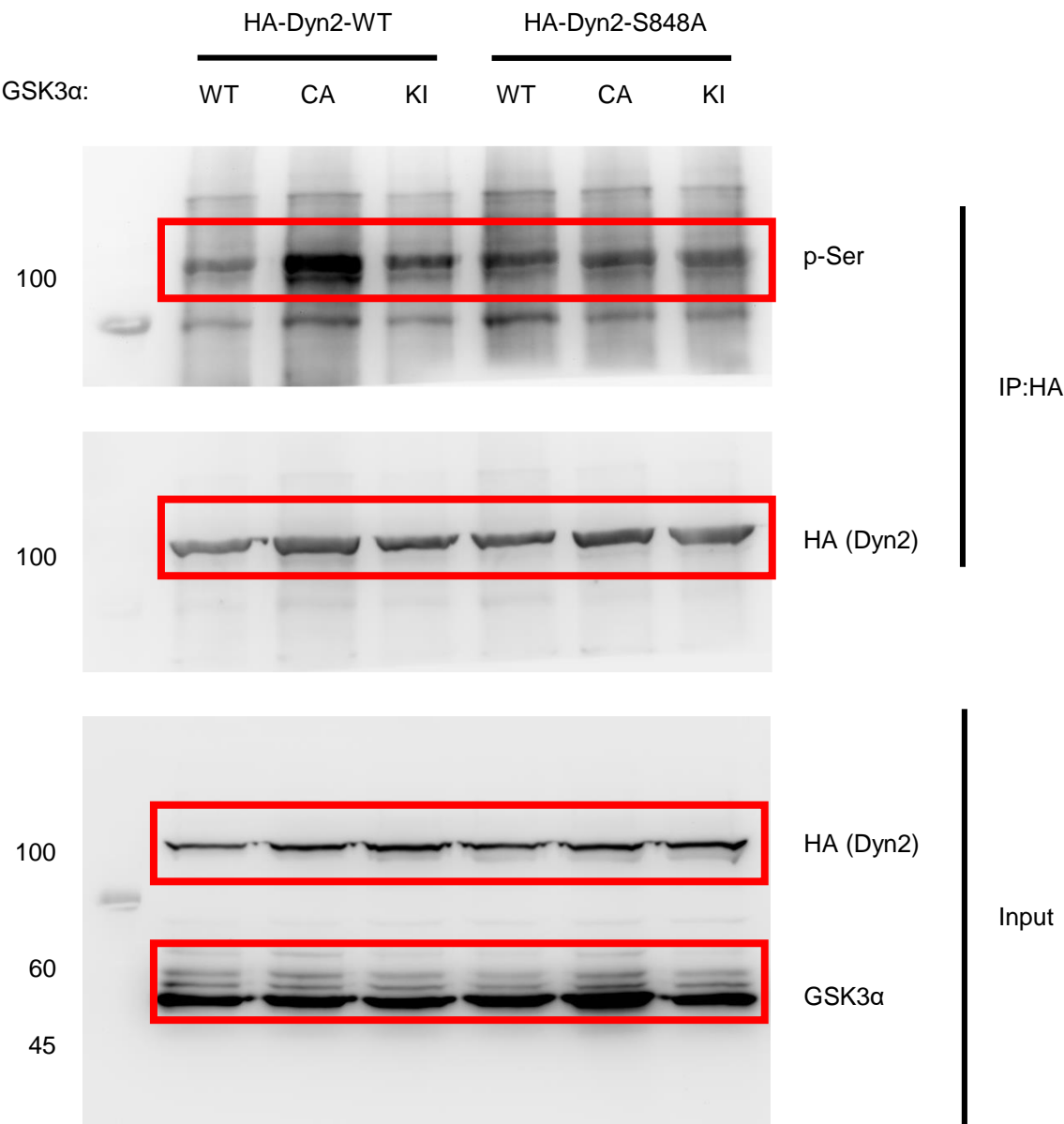

Fig 6C

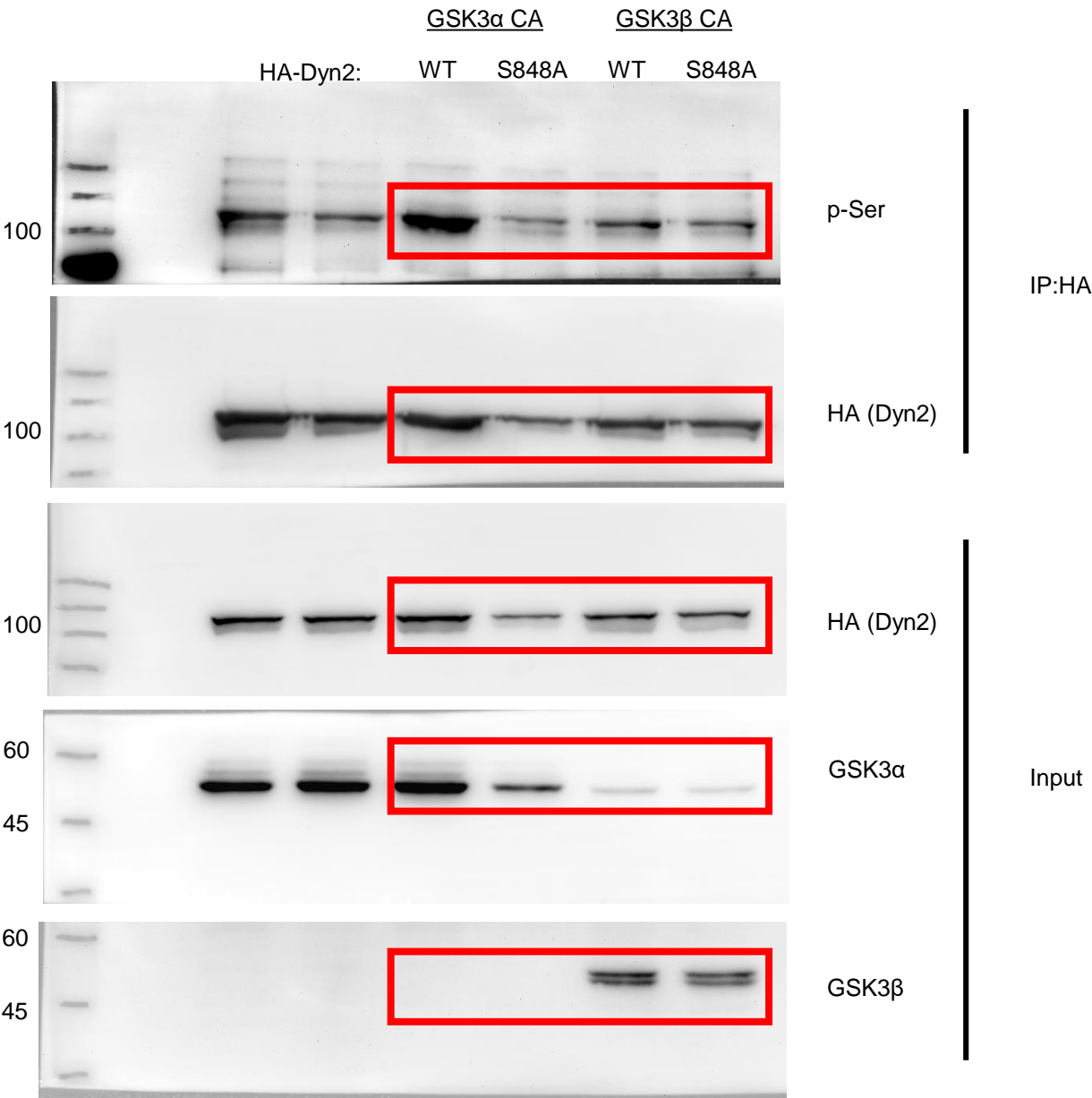

Fig 6E

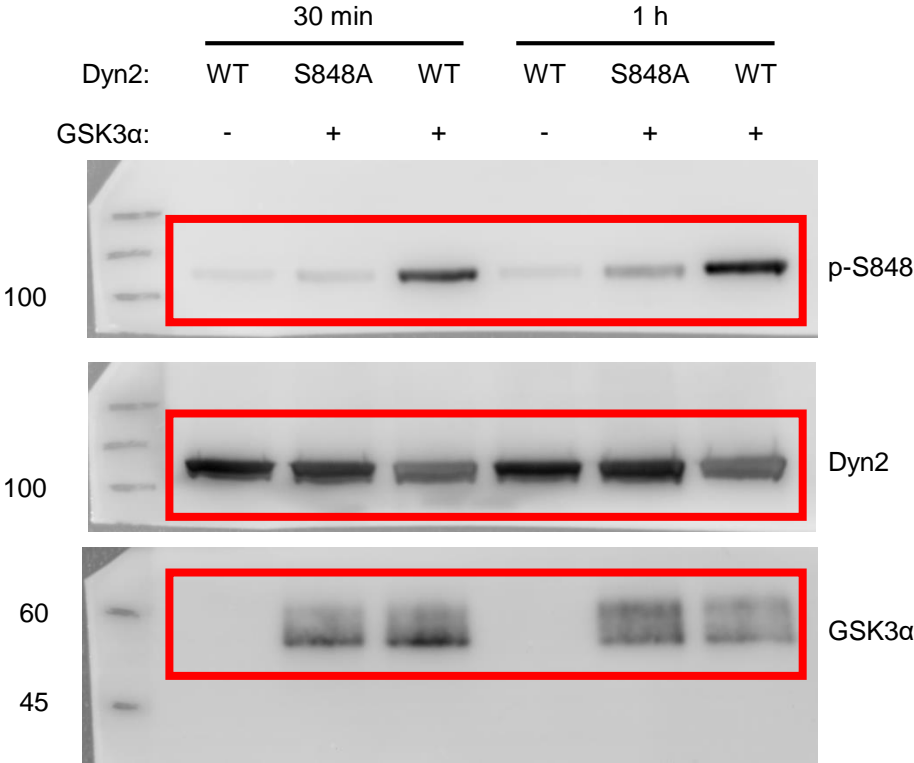

Supplement: SourceData F6 — contains original blots for Fig. 6. [file JCB_202102119_SourceDataF6.pdf]

**Fig S2A**

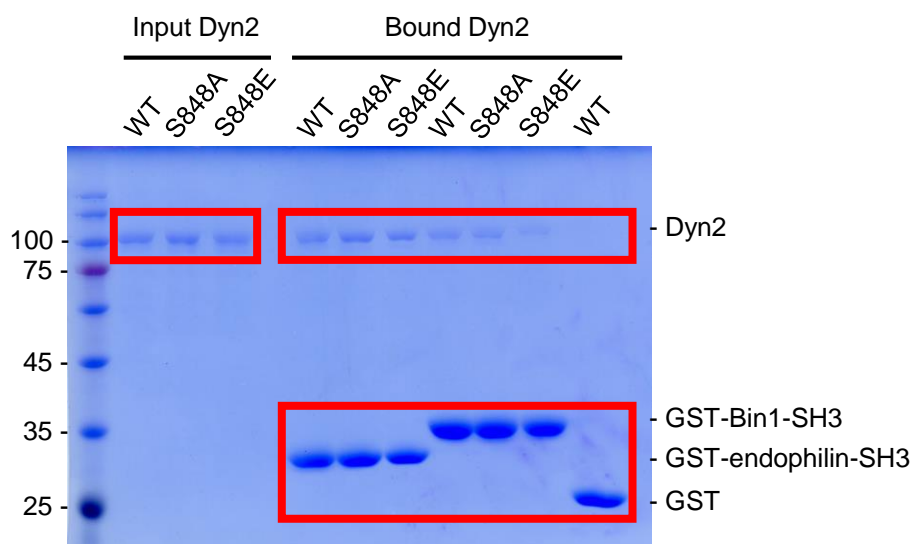

Supplement: SourceData FS2 — contains original blots for Fig. S2. [file JCB_202102119_SourceDataFS2.pdf]

Fig S3A

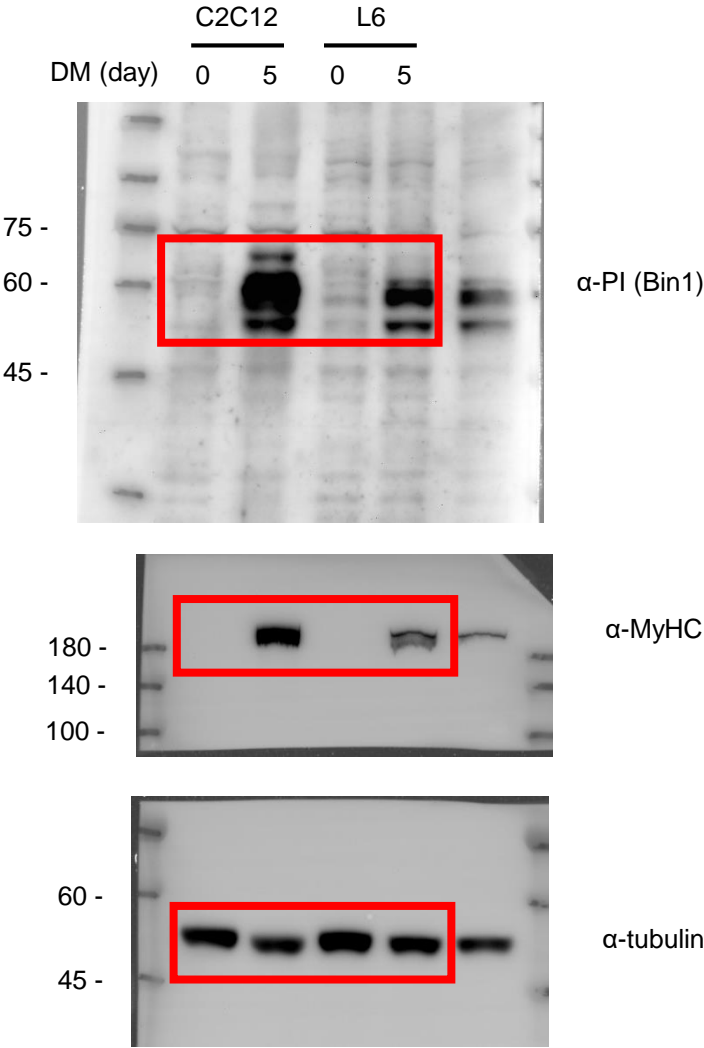

Fig S3B

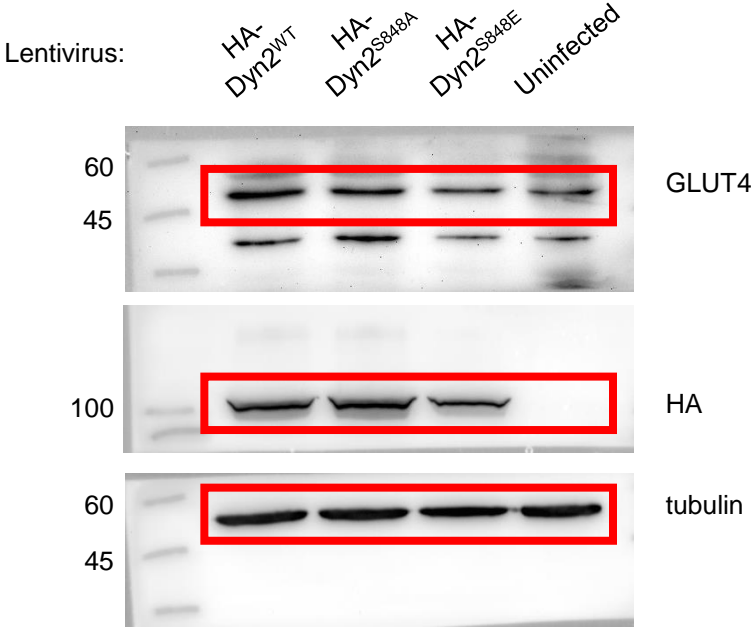

Supplement: SourceData FS3 — contains original blots for Fig. S3. [file JCB_202102119_SourceDataFS3.pdf]

Fig S4B

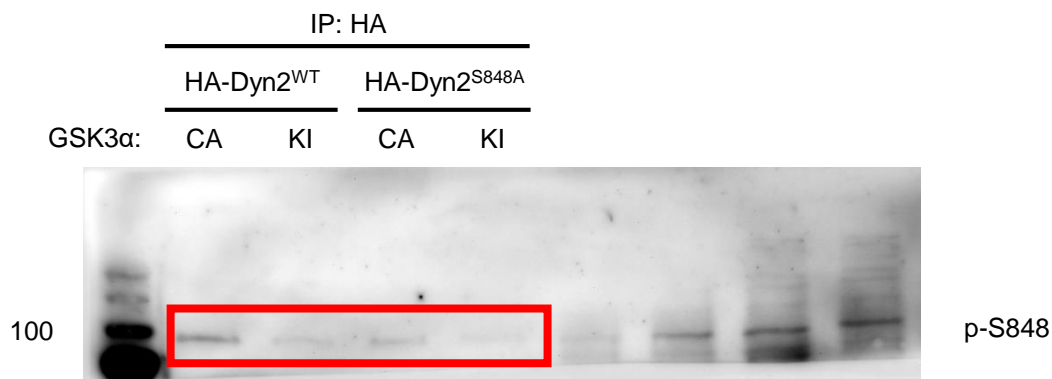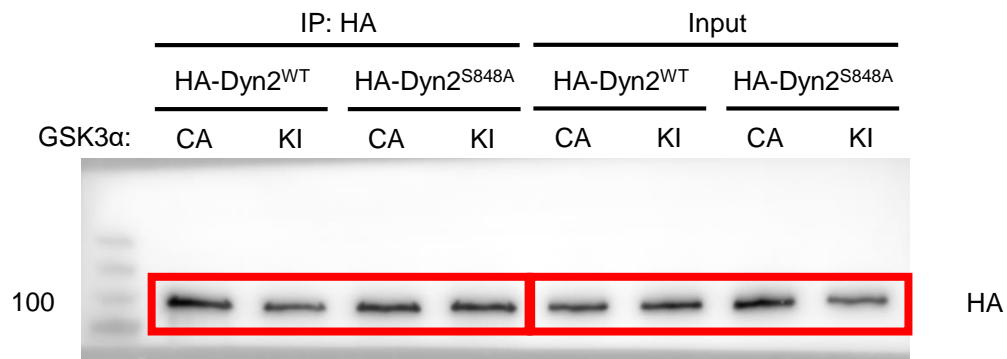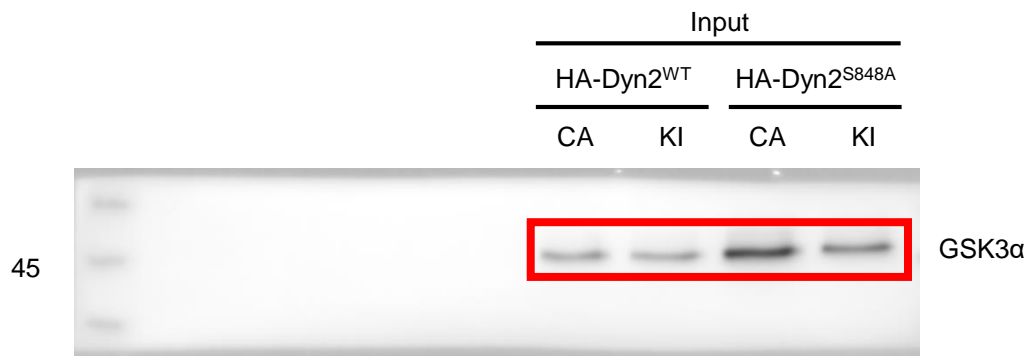

Fig S4D

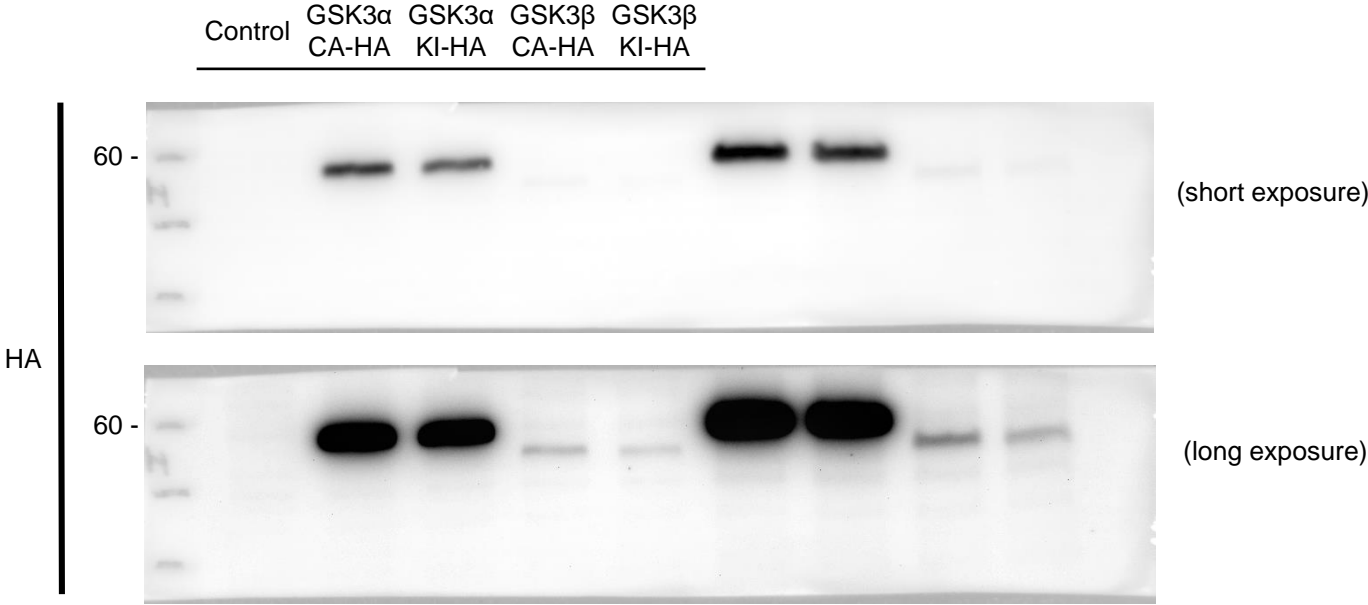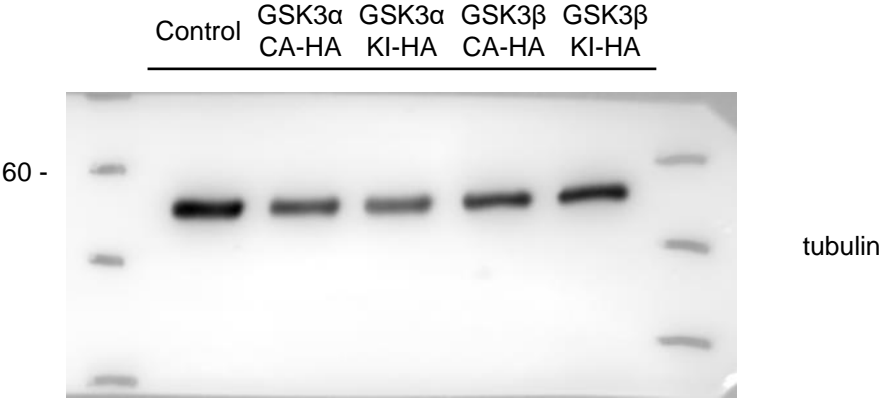

Supplement: SourceData FS4 — contains original blots for Fig. S4. [file JCB_202102119_SourceDataFS4.pdf]
