# Supplementary material for: GSK3α phosphorylates dynamin-2 to promote GLUT4 endocytosis in muscle cells
Source: J Cell Biol. 2022 Nov 29;222(2):e202102119. doi: 10.1083/jcb.202102119 (PMC9712776; doi:10.1083/jcb.202102119)
Supplement: SourceData F8 — contains original blots for Fig. 8. [file JCB_202102119_SourceDataF8.pdf]

Fig 8E

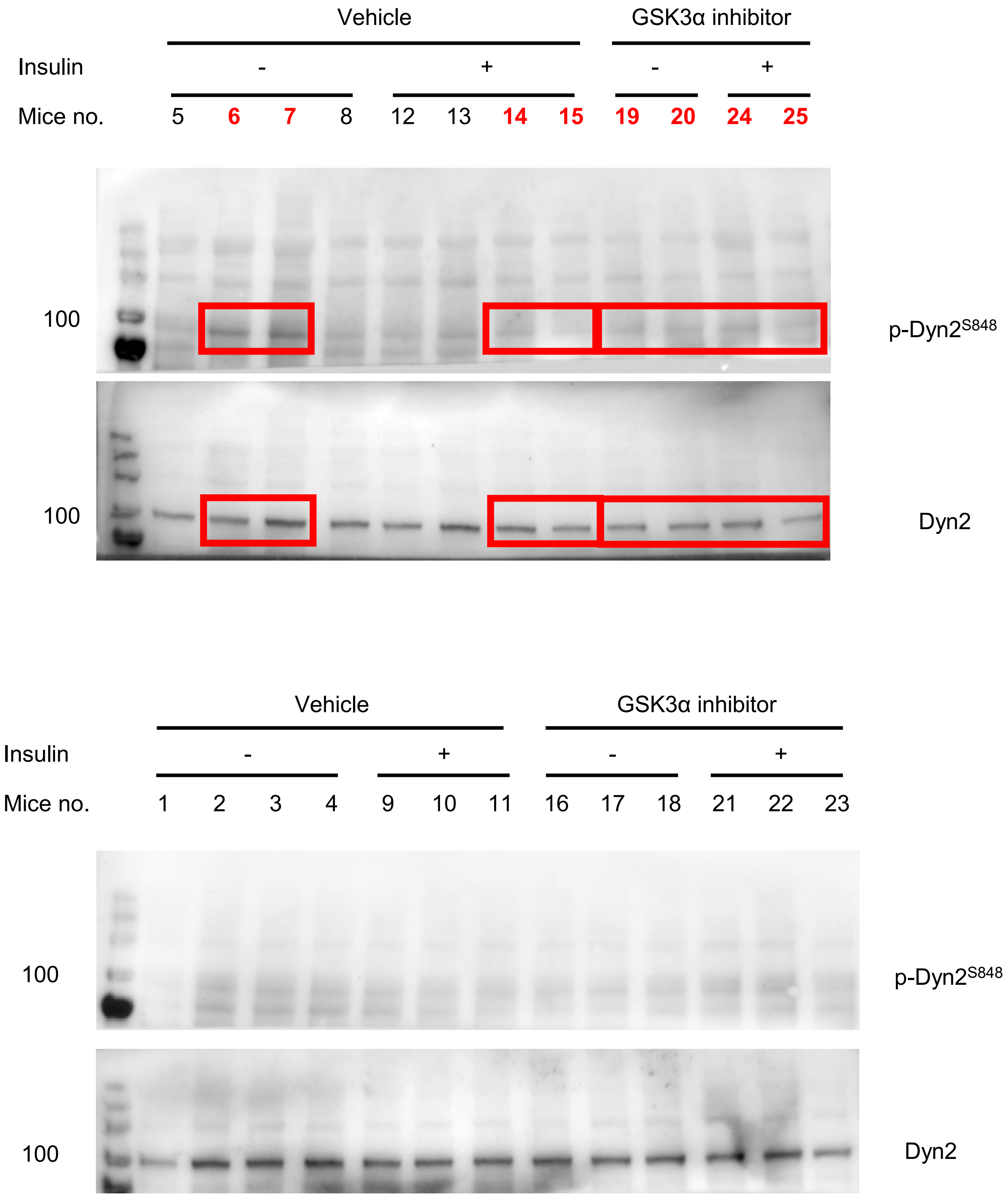

	Vehicle		GSK3α inhibitor	
Insulin	-	+	-	+
Mice no.	1-8	9-15	16-20	21-25

Fig 8F

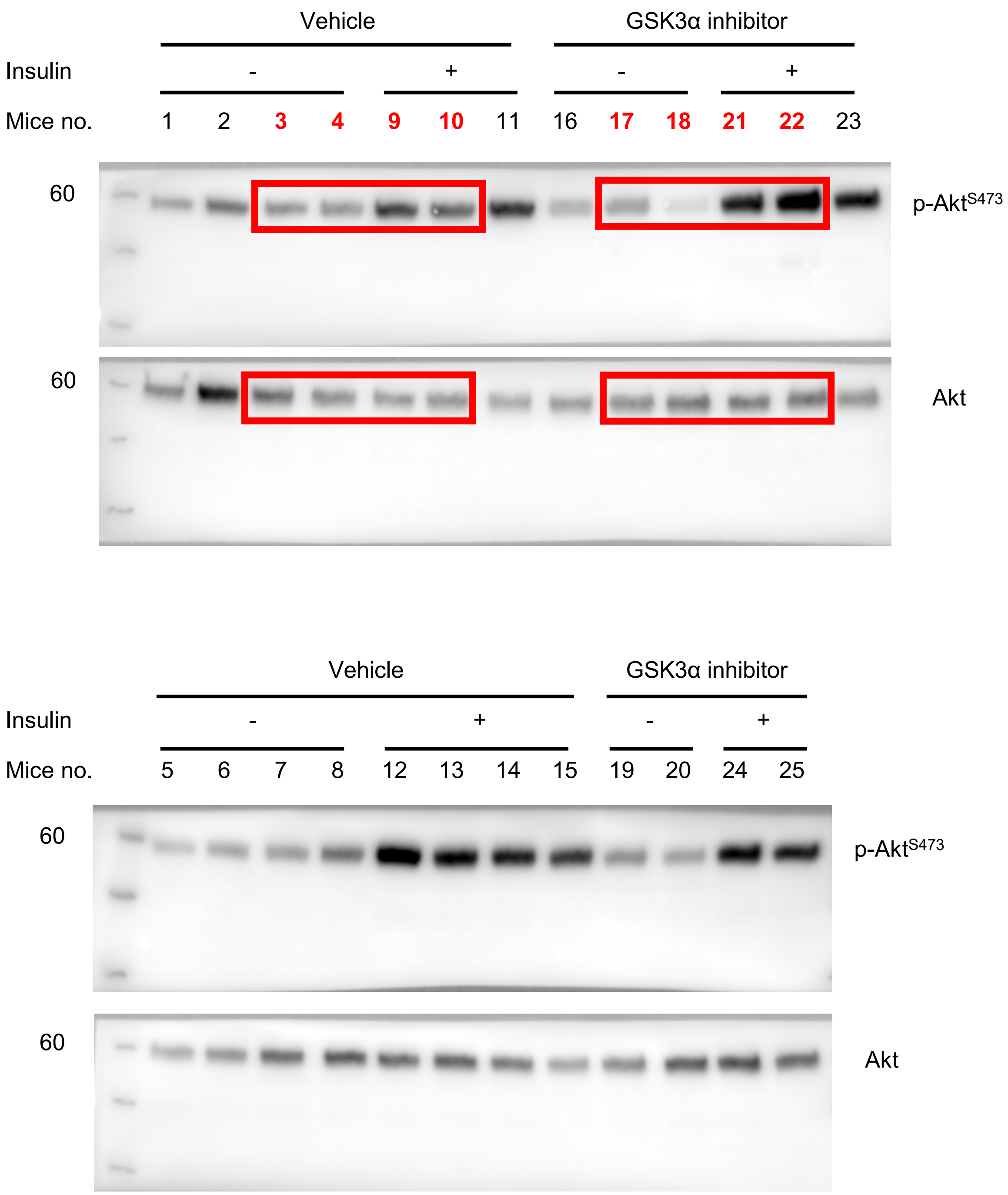

	Vehicle		GSK3α inhibitor	
Insulin	-	+	-	+
Mice no.	1-8	9-15	16-20	21-25

Fig 8G

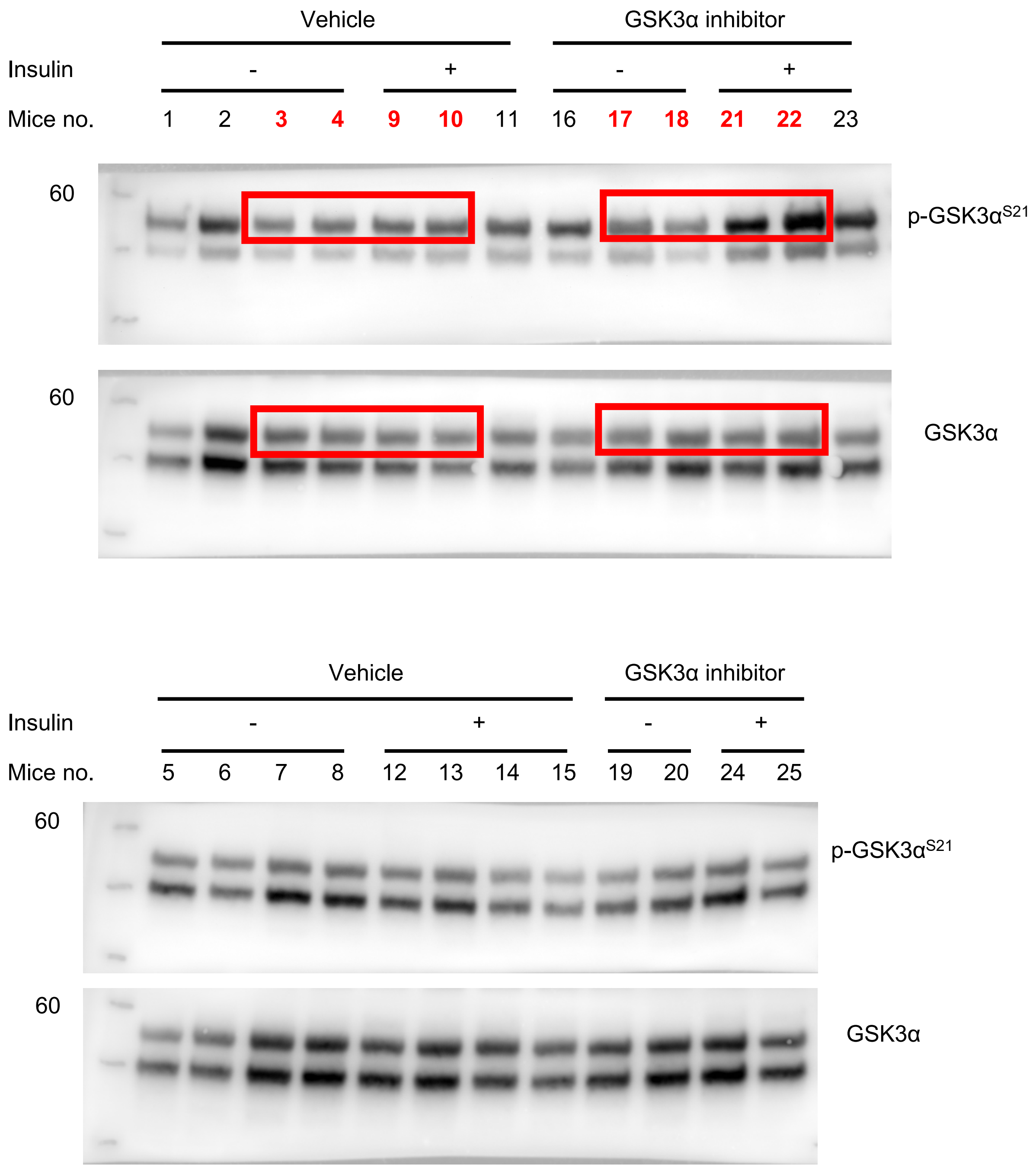

	Vehicle		GSK3α inhibitor	
Insulin	-	+	-	+
Mice no.	1-8	9-15	16-20	21-25

Fig 8H

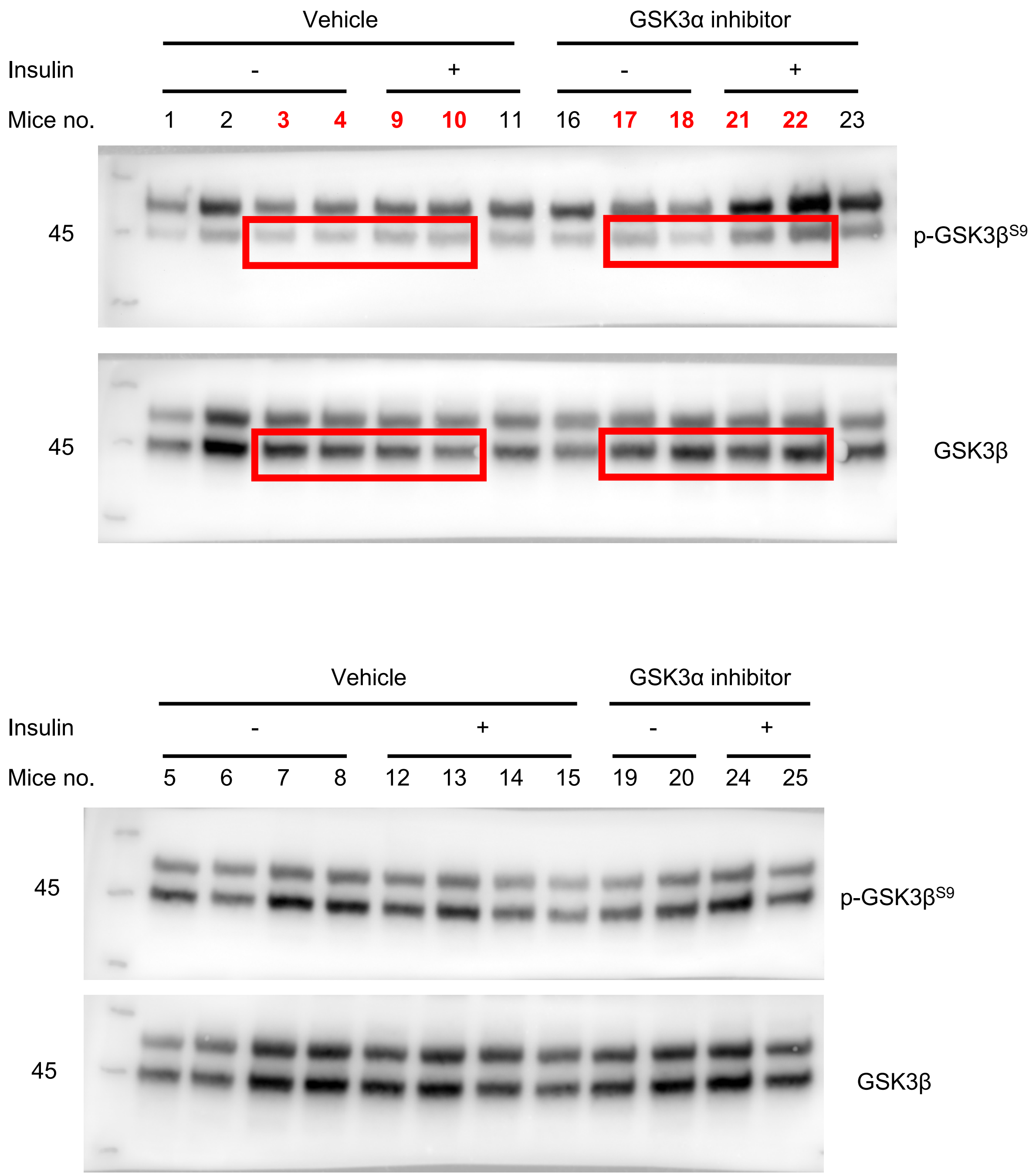

	Vehicle		GSK3α inhibitor	
Insulin	-	+	-	+
Mice no.	1-8	9-15	16-20	21-25
